# Perceived barriers and facilitators of initiation of behavioral weight loss interventions among adults with obesity: a qualitative study

**DOI:** 10.1186/s12889-018-5795-9

**Published:** 2018-07-11

**Authors:** Megan A. McVay, William S. Yancy, Gary G. Bennett, Seung-Hye Jung, Corrine I. Voils

**Affiliations:** 1 Department of Health Education and Behavior, College of Health and Human Performance, University of Florida, PO Box 118210, Gainesville, FL 32611 USA; 2 Department of Medicine, Division of General Internal Medicine, Duke University Medical Center, 501 Douglas Street, Duke Diet & Fitness Center, Durham, NC USA; 3 Department of Psychology and Neuroscience, Duke University, Box 90086, Durham, NC 27708 USA; 4 Duke Global Health Institute, Duke University, 310 Trent St, Durham, NC 27710 USA; 5 Duke Office of Clinical Research, Duke University Medical Center, 2424 Erwin Rd, Durham, NC 27705 USA; 6 William Middleton Memorial Veterans Hospital, 2500 Overlook Terrace, Madison, WI 53705 USA; 7 Department of Surgery, Division of General Surgery, University of Wisconsin School of Medicine and Public Health, K6/100 Clinical Science CEnter, 600 Highland Ave, Madison, WI 53792 USA

**Keywords:** Behavioral weight loss intervention, Intervention engagement, Intervention initiation, Qualitative research

## Abstract

**Background:**

Evidence-based behavioral weight loss interventions are under-utilized. To inform efforts to increase uptake of these interventions, it is important to understand the perspectives of adults with obesity regarding barriers and facilitators of weight loss intervention initiation.

**Methods:**

We conducted a qualitative study in adults with obesity who had recently attempted weight loss either with assistance from an evidence-based behavioral intervention (*intervention initiators*) or without use of a formal intervention (*intervention non-initiators*). We recruited primary care patients, members of a commercial weight loss program, and members of a Veterans Affairs weight loss program. Intervention initiators and non-initiators were interviewed separately using a semi-structured interview guide that asked participants about barriers and facilitators of weight loss intervention initiation. Conversations were audio-recorded and transcribed. Data were analyzed with qualitative content analysis. Two researchers used open coding to generate the code book on a subset of transcripts and a single researcher coded remaining transcripts. Codes were combined into subthemes, which were combined in to higher order themes. Intervention initiators and non-initiators were compared.

**Results:**

We conducted three focus groups with participants who had initiated interventions (*n* = 26) and three focus groups (*n* = 24) and 8 individual interviews with participants who had not initiated interventions. Intervention initiators and non-initiators were, respectively, 65% and 37.5% white, 62% and 63% female, mean age of 55 and 54 years old, and mean BMI of 34 kg/m^2^. Three themes were identified. One theme was *practical factors*, with subthemes of reasonable cost and scheduling compatibility. A second theme was *anticipated effectiveness of intervention,* with subthemes of intervention content addressing individual needs; social aspects influencing effectiveness; and evaluating evidence of effectiveness. A third theme was *anticipated pleasantness of intervention*, with subthemes of social aspects influencing enjoyment; anticipated dietary and tracking prescriptions; and identity and self-reliance factors. Different perspectives were identified from intervention initiators and non-initiators.

**Conclusions:**

Strategies to engage individuals in evidence-based weight loss interventions can be developed using these results. Strategies could target individuals’ perceived barriers and benefits to initiating interventions, or could focus on refining interventions to appeal to more individuals.

**Electronic supplementary material:**

The online version of this article (10.1186/s12889-018-5795-9) contains supplementary material, which is available to authorized users.

## Background

Lifestyle interventions that include a focus on dietary change, increasing physical activity, and behavioral strategies can produce clinically significant weight loss and improve quality of life [1–3] and therefore are recommended in current obesity guidelines [4]. However, nationally representative data suggest that only a small portion of adults with obesity (< 10%) use weight loss interventions [5]. Even in settings where interventions are readily available at low or no cost, a large majority of eligible individuals do not take the first step of initiating weight loss interventions [6–8]. Low initiation of evidence-based weight loss interventions limits the ability of these interventions to impact population-level weight [9, 10].

To increase initiation of evidence-based weight loss interventions among adults with obesity, facilitators of and barriers to initiating interventions must be identified. There is substantial literature focused on attrition from weight loss interventions [11, 12], however, this literature is of limited value for addressing intervention initiation because the factors that lead to intervention initiation may be different from those that lead to discontinuation. Indeed, several theoretical models of behavior change differentiate between factors involved in initiation and maintenance of behavior [13, 14]. For example, Rothman suggests that initiation of a health behavior is determined primarily by favorable expectations about outcomes of behavior change, whereas maintenance of behavior is determined by satisfaction with achieved outcomes [13]. Another body of literature focuses on the facilitators of and barriers to engaging in the weight loss behaviors of dietary change and exercise (independent of intervention use). However, this literature is limited in its ability to address reasons for initiating weight loss interventions; joining a weight loss intervention involves many elements in addition to dietary change and exercise (e.g., group meetings, prescriptions to track food) and therefore likely has distinctive barriers and benefits.

We are aware of three studies that specifically address barriers to weight loss intervention initiation [15–17]. All asked participants to endorse items from a list of potential barriers to intervention use. These studies revealed some potentially important intervention barriers, such as cost, stigma, concerns about group format, and concerns about intervention effectiveness. However, because these studies used close-ended surveys, they did not provide participants the opportunity to identify barriers outside of those queried about by the researchers; thus, it is possible that important barriers are missing from this research. The use of close-ended surveys in these studies also limits the details available about the identified barriers, decreasing the usefulness of this information for developing approaches to address low treatment initiation. For example, one study identified negative feelings towards interventions as a barrier to intervention use, but did not specify the nature or source of those negative feelings [15]. Additionally, these previous studies did not focus on the potential benefits of interventions. Obtaining information on what adults with obesity see as the benefits of interventions will be crucial to inform strategies to increase intervention initiation among this population. Thus, in the current study, we aimed to describe the range of reasons that adults with obesity provide for initiating or not initiating evidence-based behavioral weight loss interventions. To do this, we conducted focus groups with individuals who had recently chosen to try to lose weight with the assistance of evidence-based behavioral interventions (“intervention initiators”) or without the assistance of any formal intervention (“intervention non-initiators”).

## Methods

### Design, setting, population, and recruitment

We used a stratified purposeful sampling approach. We conducted one-time focus groups with intervention initiators who had chosen to initiate behavioral weight loss interventions in the past year and separate groups with intervention non-initiators who had not used any weight loss interventions for at least the past 5 years. Focus groups were used because the topic was initially perceived not to be sensitive in nature, and because focus groups offer greater convenience. We additionally conducted individual interviews with intervention non-initiators to allow us to go in to greater depth about topics that were raised during the focus groups and to see if other themes would emerge given the additional privacy afforded by interviews. Initiator and non-initiator focus groups were conducted separately given that the different experiences of the participants with regard to weight loss interventions necessitated that some questions differ between the two groups.

Participants were recruited from an academically-affiliated primary care clinic; a commercial weight loss intervention center (Weight Watchers); and the VA-based weight loss intervention program called MOVE!, all in Durham, NC. Intervention non-initiators were recruited from the primary care setting and intervention initiators from all three settings. Weight Watchers and the VA MOVE! program were selected as recruitment sites because they are evidence-based programs [6, 18, 19] that represent different intervention approaches and cater to different populations. Evidence-based intervention was defined using the American College of Cardiology/American Heart Association Task Force 2013 guidelines for the management of overweight and obesity in adults [4]. In particular, we considered an intervention to be evidence-based if it contained components focused on diet, physical activity, and behavioral strategies and was conducted by a trained interventionist and provided opportunity for repeated sessions. Among other differences between the two programs from which we recruited, participation in the VA MOVE! Weight Management Program generally required a provider referral at the time of our study, whereas Weight Watchers did not.

To identify primary care patients, electronic health records were used to extract contact information of patients who met eligibility criteria for age, BMI, and contraindications to weight loss, and who had a primary care appointment in the previous 2 months. These patients were sent an informational letter and were proactively called for further screening if they did not opt out. Participants recruited at weight loss programs were informed of the study via an announcement at the beginning of a class and were called by telephone for screening if they gave permission and contact information.

Inclusion criteria included age 18–70 and BMI ≥ 30 kg/m^2^ based on self-reported weight and height. Because we were interested in why individuals attempt weight loss with assistance from an evidence-based comprehensive intervention versus attempt weight loss on their own without formal assistance, we excluded people who had made no weight loss attempt in the past year (i.e., those who responded negatively to “Have you tried to lose weight in the past year by changing your diet or physical activity level?”). We focused on the past year so that participants would be more likely to recall their experience of choosing to use or not use a weight loss program. Exclusion criteria included conditions contraindicated for weight loss (e.g., pregnancy, cancer in past year) or bariatric surgery in past year. An additional inclusion criterion for intervention initiators was that they had used an evidence-based formal weight loss intervention in the past year (with evidence-based intervention defined according to clinical guidelines presented above). Individuals were eligible if they used a group or individual format interventions. Intervention non-initiators were required to have no use of evidence-based formal interventions in the past 5 years. A five-year time frame was selected to create a clear distinction between intervention initiators and non-initiators.

### Procedures

Focus groups and individual interviews were conducted on the academic medical center campus. Group discussions lasted approximately 90 min, and individual interviews lasted about 30 min. Groups and individual interviews were moderated by the first author, who had no clinical relationship with participants. All focus groups and interviews were audio recorded and later transcribed. We planned a priori to conduct three focus groups for initiators and three for non-initiators, and then to evaluate if this number of groups was sufficient to achieve informational redundancy and conduct more as needed. At the end of each focus group and interview, two authors reviewed themes that emerged. No new themes were raised in the third wave of groups, indicating saturation had been reached. However, we elected to conduct individual interviews of non-intervention initiators after considering that participants might be reluctant to share in a focus group setting their concerns about group-based interventions. We determined that saturation was reached by the end of eight interviews and thus did not conduct more.

A semi-structured moderator script was used to guide discussion (see Additional file 1 for script). This script was developed based on a qualitative content analysis approach, a method for interpreting textual data through a systematic classification process [20]. The moderator script for initiators and non-initiators differed as a consequence of the different histories these groups had with weight loss interventions. We began the non-initiator discussions by asking what led participants to try to lose weight in the past year without intervention. We then provided a definition of evidence-based intervention. We asked them to think about evidence-based interventions delivered in a group setting and to discuss what it would take for them to join such a program as well as what concerns they might have about doing so. We chose to focus on group format interventions given that they are the most common and often most cost-effective intervention approach. We also asked about telephone and internet-based treatments, but that data is not presented here.

We began the initiator groups by asking them to discuss what led them to join a weight loss intervention. When it was not spontaneously addressed, we probed about the role of learning more about diet, physical activity, social support, and motivation. We also asked the initiator groups what barriers they had to overcome before joining a program.

For initiators and non-initiators, we next queried about the following specific barriers to intervention based on past research in the areas of behavioral intervention initiation, weight management more generally, and the authors’ experiences: embarrassment discussing weight, interacting with others, time/scheduling conflicts [16, 17], cost [17], support from family/friends [21], intervention asking them to do things they do not wish to do (self-monitoring, exercising more than they want to) [22], and who would lead the intervention. The moderator guide was created by two study authors and was reviewed by other study authors. We pilot-tested the guide with a small focus group (data not analyzed) and refined the interview guide by re-wording and re-ordering questions before collecting the data reported herein.

### Data analysis

A conventional content analysis approach was used to analyze the data [20]. Focus group and individual data were analyzed with the same approach and presented together, consistent with the study goal of generating as many perspectives as possible. All transcripts were placed into qualitative data management software (Atlas.ti, Version 7.5). Two coders with backgrounds in social and clinical psychology independently coded the first transcript using open coding, allowing codes to emerge from the data, then compared and reconciled codes. They repeated this procedure for approximately 60% of the transcripts in order to develop a taxonomy of consensus codes. The coding scheme was then applied to the remaining transcripts by a single coder. After reviewing codes, both coders identified subthemes that emerged from the codes and higher-order themes emerging from the subthemes. A matrix was created to compare presence of specific codes by intervention initiation status for each subtheme. Quotes in this manuscript (provided in tables) were chosen to be illustrative of the subthemes.

## Results

### Participants

Three intervention initiator groups were conducted with 24 total participants (group sizes: 5, 9, and 10). Three intervention non-initiator groups were conducted with 26 total participants (group sizes: 6, 9, and 11). In addition, 8 non-initiator participants completed individual interviews. Thus, there were 58 participants in total (24 intervention initiators and 34 non-initiators).

Participants had a mean age of 54–55 years, mean BMI of 34 kg/m^2^, were predominantly female and college educated or higher (Table 1). Intervention initiators were predominantly white, whereas non-initiators were predominantly African American (Table 1).

**Table 1:** Demographic and weight history characteristics of intervention initiators and intervention non-initiators

	Intervention Initiators (*n* = 26)	Intervention non-initiators (*n* = 32)
Sex, n (%)		
Male	10 (38.5)	12 (37.5)
Female	16 (61.5)	20 (62.5)
BMI, kg/m^2^, m (SD) ^a^	34.3 (5.7)	33.8 (3.0)
Age, m (SD)	54.5 (13.8)	54.4 (12.9)
Race, n (%)		
African American	9 (34.6)	18 (56.3)
White	17 (65.4)	12 (37.5)
Asian	0	2 (6.3)
Education, n (%)		
High school or less	2 (7.7)	3 (9.4)
Some college or tech school	9 (34.6)	12 (37.5)
Bachelor’s degree or higher	14 (53.8)	17 (53.1)
Marital status, n (%)		
Married or married-like relationship	17 (65.4)	15 (46.9)
Divorced, widowed, or single	9 (34.6)	17 (53.1)
Work status, n (%)		
Work full time	9 (34.6)	14 (43.8)
Retired/not working/working part time	17 (65.4)	18 (56.3)
Number of lifetime uses of weight loss intervention, n (%)		
Never	4 (15.4)	23 (71.9)
1–5	20 (76.9)	6 (18.8)
6 or more	2 (7.7)	3 (9.4)

BMI based on self-report

^a^*n* = 25 for BMI for initiators and *n* = 31 for non-initiators

The perspectives on barriers and benefits of initiation of evidence-based weight loss interventions were classified into three higher-order themes, each with several subthemes.

### Practical factors

One higher-order theme identified was practical factors. This theme included the subthemes of reasonable costs and scheduling compatibility (See Table 2 for summary).

**Table 2:** Summary of factors favoring treatment initiation and favoring non-initiation included in *Practical Factors* theme

	Both Initiators and Non-initiators	Initiators only	Non-Initiators only	Representative Quote
Reasonable cost	Favoring initiation • Able to afford • Paid for by work or insurance	Favoring initiation • Money saved by changing eating habits offset program costs	Favoring initiation • Cost is reasonable for expected effectiveness Favoring non-initiation • Not able to afford	“Cost would be a factor. I would be less likely to spend a lot of money on something unless I was really guaranteed what the result was.” (non-initiator)
Scheduling compatibility	Favoring initiation • Compatibility with other obligations • Flexible schedule/drop-in options Favoring non-initiation • Anticipate incompatibility with other obligations	Favoring initiation • Short sessions	NA	“Whatever the time and date would be, I have to miss half of them because of traveling or work or whatever it is.” (non-initiator)

#### Reasonable costs

Both intervention initiators and non-initiators reported that intervention entry was facilitated by having the ability to afford an intervention, either on their own or through insurance or workplace programs. Intervention initiators, but not non-initiators, reported that intervention entry was favored as a result of money saved due to changing their eating habits as a result of joining the intervention (e.g. via reducing restaurant eating) offsetting the costs of the intervention. Intervention non-initiators indicated that if the cost appeared reasonable in relation to the expected effectiveness, intervention entry would be facilitated. Non-initiators also indicated that being unable to afford the intervention was a potential barrier to intervention entry.

#### Scheduling compatibility

Both intervention initiators and non-initiators reported that compatibility of the intervention schedule with other obligations favored initiation, as did flexibility in scheduling intervention sessions (e.g., availability of classes at different times of day without the requirement to commit to specific class). For both initiators and non-initiators, anticipation of incompatibility with their schedule favored non-initiation. Intervention non-initiators stated that short intervention sessions would be a facilitator to intervention entry.

### Anticipated effectiveness of intervention

The second theme was anticipated effectiveness of intervention. Individuals’ decisions to initiate interventions were influenced by their perceptions of how effective it would be in helping them achieve weight loss. Subthemes identified that contributed to their perceptions of intervention’s anticipated effectiveness were: 1) how the content of interventions would address their needs, 2) how social aspects of interventions enhance intervention effectiveness, and 3) their evaluation of the evidence of interventions’ effectiveness (See Table 3 for summary).

**Table 3:** Summary of factors favoring treatment initiation and favoring non-initiation included in *Anticipated Effectiveness of Treatment* theme

	Both Initiators and Non-initiators	Initiators only	Non-Initiators only	Representative Quote
Treatment’s content addresses needs	Favors initiation • Offers behavioral strategies • Offers nutritional information	Favors initiation • Physical activity not a major focus • Offers individualized information	Favors initiation • Offers a focus on physical activity Favors non-initiation • Content would not be individualized or relevant to them • Would not offer anything new	“I live alone, I’m not taking care of family, and I’m not cooking for other people. If 70 to 80% of the time in the group was dealing with strategies for scheduling things, that would not be good for me.” (Non-initiator)
Treatment’s social aspects enhance perceived effectiveness	Favors initiation • Group or leader: ○ provides support/affirmation ○ provides accountability ○ provides learning opportunities • Group provides competition	NA	NA	“Walking in and seeing and talking to those ladies that are there and getting on that scale and they always say ‘remember you don’t always have to get on the scale.’ [I say:] ‘Yes I do.’ That’s the part I need is that check-in once a week.” (Initiator)
Evaluation of evidence for effectiveness	Favoring initiation • Has scientific evidence base • Treatment recommendation from trusted health care provider • Learning of others having success with treatment • Personal past poor outcomes of weight loss efforts without treatment	Favoring initiation • Longevity of treatment’s existence enhance credibility claims • Past personal success with treatment	Favoring initiation • Evidence from credible source. • Offering a trial period to evaluate effectiveness Favoring non-initiation • Skepticism of evidence for effectiveness • Learning of others having poor outcomes with treatment • Past personal experience of poor outcomes with treatment. • Past success with weight loss without treatment	“I trust her (my doctor) with my life, and I go to her, and she checks my insides and helps me when I am sick get better. So, I would [go to weight loss treatment] if she asked or suggested it and gives me a program. Evidently she has done enough to know enough and practiced enough to know what might be good for me.” (Non-initiator).

#### Intervention’s content addresses needs

Both intervention initiators and non-initiators reported that intervention entry was facilitated by the desire to learn behavioral strategies and a desire to gain useful information about nutrition. Intervention initiators reported that their decision to enter an intervention was positively influenced by the intervention they selected having a limited focus on physical activity. On the other hand, non-initiators identified a focus on physical activity as favoring intervention initiation. They shared their perception that physical activity is important to weight loss; some reported believing that physical activity alone was enough to lose weight.

Both initiators and non-initiators commented on the individualization of intervention content, but from different perspectives. Initiators identified individualization as a factor that favored their entry in to intervention. For example, an intervention initiator reported that the ability to obtain personalized dietary suggestions contributed to her decision to initiate an intervention (notably, she was enrolled in a one-on-one program). For intervention non-initiators, concern that content would not be individualized or match their specific life situation was identified as favoring non-initiation of interventions. Related to this, non-initiators expressed the perception that interventions would not offer anything that was not already known to them, or that they could not learn or do on their own.

#### Intervention’s social aspects enhance perceived effectiveness

Some intervention initiators and non-initiators felt that a group format enhances weight loss success through support and affirmation from others who are having similar experiences. Both initiators and non-initiators also reported that having a group or an intervention leader also facilitated intervention entry by offering accountability, e.g., intervention leaders and/or group members who would be aware of and track their successes and struggles. Initiators and non-initiators also reported that a group format could offer learning opportunities, such as when group members share their weight loss strategies or recipes. Additionally, both initiators and non-initiator participants reported that a group-based weight loss program would be appealing to them because they could motivate themselves by viewing weight loss as a competition with other group members.

#### Evaluating evidence of effectiveness

Intervention initiators and non-initiators reported that learning of a scientific evidence base for an intervention would increase their trust in a intervention’s effectiveness, and thus would favor intervention initiation. Both groups also reported that intervention initiation would be favored by obtaining a recommendation to join intervention by a trusted health care provider. Intervention initiators and non-initiators described how observing others’ experiences with weight loss and their own past experience with weight loss efforts influenced their perception of initiating an intervention, with some difference in these observations between initiators and non-initiators (see Table 3 for additional details).

Intervention initiators, but not non-initiators, reported that intervention entry was favored when a weight loss intervention program had been operating for a prolonged period, thus increasing their confidence in its effectiveness. Non-initiators reported that entry to interventions was negatively influenced by skepticism of some claims made by interventions, particularly when the presenter of the claims may have motives to be dishonest, such as a desire to profit from an intervention.

### Anticipated pleasantness of interventions

The third theme was anticipated pleasantness of interventions. The decision to enter an intervention was reported as being influenced by perceptions of how pleasant or unpleasant the experience of the intervention would be. Subthemes identified that contributed to the perceived pleasantness included 1) social aspects influencing pleasantness, 2) intervention’s dietary and tracking prescriptions, and 3) identity and view of self-reliance as it relates to intervention use (see Table 4 for summary).

**Table 4:** Summary of factors favoring treatment initiation and favoring non-initiation included in *Anticipated Pleasantness of Treatment* theme

	Both initiators and non-initiators	Initiators only	Non-Initiators only	Representative Quote
Social aspects influencing enjoyment	Favoring initiation • Starting treatment with friend/family member Favoring non-initiation • Concern about disappointing others or feeling judged	Favoring initiation • Use strategies to manage anxiety Favoring non-initiation • Frustration with input of some group members • Competition can be intimidating, discouraging	Favoring non-initiation • Concern about relating to group members • Concern about disclosing sensitive information in group	“I didn’t want to talk. The one thing I worried about when you go to these meetings is, will it go bad if you don’t talk and are you required to talk? So I talked the instructor before I signed up and she was like, ‘I will not make you talk.’” (Initiator)
Anticipated dietary and monitoring recommendations	Favoring non-initiation • Treatment approach requiring eating prescribed foods or meals	Favoring initiation • Willingness to rearrange schedule to fit food preparation demands • Treatment approach to food is compatible with family • Treatment approach to food does not contribute to feeling deprived. • Program makes tracking diet easy	Favoring initiation • Treatment approach to food is quick and easy Favoring non-initiation • Treatment requiring complicated diet tracking	“I think I am lazy. If somebody could deliver me some meals and they are already ready, I am going to eat what you put in front of me. That would help me a lot because, if I am on the move and I am coming home from work, I might stop somewhere that is convenient. Or I might cook something that’s convenient, but it is not really that healthy.” (Non-initiator)
Identity and self-reliance factors	Favoring non-initiation • Embarrassed to need help	Favoring initiation • Accepting that they cannot do it on own	Favoring non-initiation • Desire to not rely on others for weight loss • Desire to avoid loss of autonomy by a program • Treatment use not compatible with identity	“I mean that’s for big fat people, and I’m not like that.” (non-initiator)

#### Social aspects influencing enjoyment

Both intervention initiators and non-initiators reported that starting an intervention alongside someone they know could make initiating intervention more pleasant, favoring intervention entry. Although both initiators and non-initiators described positive aspects of accountability (discussed above with regard to intervention effectiveness), participants in both groups also reported that accountability could be a barrier to intervention initiation by impacting their anticipated enjoyment of an intervention. Specifically, they reported that having others monitoring their progress presented a risk of disappointing others, causing them embarrassment, and feeling judged.

Intervention initiators, but not non-initiators, reported that intervention initiation was facilitated by use of strategies to manage anxiety related to the social aspects of the group. Examples included using a “pep talk” to manage anxiety about group setting, purposefully selecting a program that they believed would have a non-judgmental approach, and selecting a program that did not require them to talk in a group format. For initiators, a potential barrier to initiation was frustration with the interactions with some group members, for example, individuals who dominate the group discussion or who share things that are off-topic. Also favoring non-initiation among intervention initiators was concern about competition among group members, which some participants felt would be intimidating and discouraging (in contrast to other members who found competition to favor intervention entry due to possible increase in intervention effectiveness, noted previously).

Barriers to intervention entry reported by intervention non-initiators include not relating to other group members and concern that they would have to share sensitive or potentially embarrassing information in a group setting (e.g., discussing gastrointestinal issues).

#### Anticipated dietary and monitoring prescription

Both initiators and non-initiators reported that an intervention that prescribed specific foods that they disliked, or that required foods to be eaten at certain times (e.g. requiring breakfast), would be a barrier to intervention entry. Intervention initiators, but not non-initiators, reported that intervention initiation was facilitated by a willingness to rearrange their personal schedule to fit demands of food preparation, such as coming home from work early to prepare food. Initiators also reported that intervention entry was facilitated by an approach to food that was compatible with their family life (e.g., that allowed them to share meals with their family). Finally, intervention initiators reported that initiation was favored when interventions offered an approach to food that would not contribute to feeling deprived, for example, by allowing them to continue to eat their favorite foods.

Non-initiators reported that intervention entry would be favored if interventions had an approach to food that was quick, easy, and convenient (e.g., pre-packaged food). Non-initiators also reported that a barrier to intervention entry was anticipating that the intervention would require tracking food, and particularly, that it would require a complicated approach to tracking. Related to this, intervention initiators reported that an approach to dietary tracking that is simple and easy favored intervention entry.

#### Identity and self-reliance factors

Both initiators and non-initiators reported that a barrier to intervention entry was embarrassment that they could not lose weight on their own. Related to this, intervention initiators reported that intervention entry was facilitated when they accepted that they needed help losing weight. Intervention non-initiators reported that another barrier to initiating an intervention was a desire to avoid reliance on others and concerns about having their autonomy compromised. Additionally, non-initiators described concerns that intervention initiation would be inconsistent with their identity.

## Discussion

Effective interventions to help individuals lose weight are underutilized. Only a few previous studies have examined the perspectives of adults with obesity on barriers and benefits to weight loss intervention initiation [15–17], and those studies queried participants about a narrow range of possible barriers to intervention use and did not address potential benefits of intervention use. In the current study, we aimed to describe the breadth of benefits and barriers to initiating evidence-based weight loss interventions as perceived by a diverse sample of adults with obesity who have recently tried to lose weight, either with or without the help of a formal weight loss intervention. Our data revealed several novel facilitators and barriers to intervention initiation and identified potential differences in perspectives between intervention initiators and non-initiators.

Reasons for intervention initiation reported by participants were aggregated in to the broad themes of practical factors, anticipated intervention effectiveness, and anticipated pleasantness of interventions. The theme *anticipated effectiveness of intervention* related to participants’ sense for how well an intervention would work for them, and the factors that influenced that. This theme has some conceptual overlap with constructs from several theoretical models, including *perceived benefits of health behavior* from the Health Belief Model [23] and *outcome expectancies* from Social Cognitive Theory [24]. Anticipated effectiveness was also suggested as a determinant of intervention initiation by past research. Specifically, Tinker and Tucker found that the most highly cited barrier to intervention initiation was the belief that individuals could lose weight as well on their own as with an intervention [15]. However, that study, and existing theory, do not provide information on what factors influence perceptions of effectiveness. In this study, we found that intervention initiation is favored when content is perceived to be individualized and addresses individuals’ perceived needs; when social aspects of an intervention that are perceived to increase intervention effectiveness are present (such as a leader to increase accountability); and when sources of evidence for an intervention’s effectiveness are trustworthy (such as a recommendation by a health care provider). There was some evidence of a potential difference between initiators and non-initiators in perceptions of intervention effectiveness. Of particular note, non-initiators were concerned that intervention would not be individualized or would not offer them any new information, whereas initiators, who predominantly enrolled in group-based interventions, did not express this concern.

*Anticipated pleasantness of interventions* also emerged as a theme from our data. This theme primarily focused on negative experiences that individuals anticipate with interventions. There is some conceptual overlap of this theme with The Health Belief Model construct *perceived barriers to health behavior*. Negative or unpleasant experiences have also been identified as a barrier to dietary change or intervention in past empirical literature. For example, Burke et al. reported that some weight loss intervention participants found self-monitoring burdensome [22] and numerous studies report that taste preferences can be a barrier to changing diet [25, 26]. Additionally, Ciao et al. [16] identified shame and stigma as barriers to weight loss intervention use in a small portion of their participants. However, the current study provides a fuller picture of the potential unpleasant experiences that concern intervention initiators and non-initiators. Unpleasant experiences of concern included discomfort with social aspects of intervention (e.g., fear of judgement), dislike of anticipated dietary recommendations, and worry about needing to disclose sensitive information in a group. Whereas past research identified that dietary tracking and eating less preferred foods could be viewed negatively [22, 25, 26], the current study is the first to our knowledge to identify the role of dietary monitoring and taste preferences as potential barriers to initiating interventions. We also found that differences emerged between intervention initiators and non-initiators related to anticipated pleasantness. For example, non-initiators reported a desire to avoid relying on others for weight loss and to maintain their autonomy, whereas initiators reported that intervention initiation was facilitated when they recognized a need to get help in order to lose weight.

We also identified *practical factors* as a theme. Practical barriers such as cost have been identified in previous studies on intervention use barriers [16, 17] and these factors are consistent with the construct *perceived barriers of health behavior* within the Health Belief Model.

These results may inform strategies for increasing initial engagement in weight loss interventions. Two distinct approaches to increasing intervention initiation can be informed by results of the current study. First, *intervention-focused approaches* involve changing some aspect of an intervention to increase the chances of initiation. For example, a barrier to intervention entry described by some of our participants was intensive dietary tracking, which is common in evidence-based programs. Interventions that offer simpler tracking approaches may be more appealing to some patients. Previous research suggests that simpler self-monitoring approaches can be effective for increasing adherence and weight loss [27, 28], suggesting that offering approaches that utilize these simpler self-monitoring methods might be valuable for increasing intervention uptake. As another example, our results suggest that some individuals are seeking a physical activity-focused approach to weight loss and are not drawn to existing interventions that primarily focus on diet. Greater intervention enrollment might result if intervention options were available that placed greater emphasis on physical activity. It is notable that some individuals’ intervention preferences, such as greater emphasis on physical activity or less dietary tracking, may make the intervention less effective. Potentially, offering interventions that are less effective than the gold-standard intervention approach (while still more effective than no intervention) could increase population weight loss if the greater uptake offsets the lower weight loss per person.

The other potential set of approaches to increasing intervention entry are *individual-focused approaches*, which focus on addressing individual barriers and facilitators to entering existing evidence-based interventions. For example, some non-initiators felt that they already knew everything that an intervention would tell them. Thus, when communicating to individuals interested in losing weight, it may be important to communicate that an intervention is valuable for providing motivation and support, rather than just knowledge. Efforts to increase initiation could involve providing education on the evidence for relatively greater importance of diet compared to physical activity for weight loss. Given the variability we observed in intervention preferences and barriers to interventions, offering several types of interventions, or interventions that can be customized on important dimensions, may lead to greater uptake.

There are several limitations to this study. Although we compared initiators and non-initiators, there were some differences in the prompts that were used between the two groups, due to differences in their history of intervention use. Our intervention non-initiator group included people who may have used an intervention more than 5 years ago; different results might be obtained with only individuals who have never used intervention. Because our interview script asked specifically about group-based interventions, our data may not have captured factors that would differentially affect the decision to enter a one-on-one intervention. Individuals not represented in our study, such as those who attended one-on-one programs, may have perspectives that we did not observe in this study.

Finally, although there is some evidence that intervention initiation differs by race and gender [29–31], this study was not designed to examine gender and racial or ethnic differences. It is notable that a few of the factors relevant to intervention initiation identified in this study differ across races in the general US population, and thus could contribute to racial differences in intervention initiation. In particular, we identified that obtaining a referral from a trusted health care provider could facilitate intervention entry; given that African Americans on average report greater distrust of health care professionals [32], lower trust may contribute to racial differences in intervention initiation. Similarly, we identified cost as a barrier to intervention entry; given mean differences in wealth between African Americans and white Americans, this cost barrier could also contribute to racial differences in intervention use.

## Conclusions

Greater initiation of weight loss interventions may be achieved by addressing the factors identified in this study among individuals who are trying to lose weight without interventions. Tailored approaches to increasing intervention initiation may provide the most benefit given observed variability in facilitators and barriers to intervention entry.

## Additional file

Additional file 1: Qualitative focus group/interview moderator script. This is the script used to moderate the focus groups and interviews conducted for this study. (PDF 348 kb)
