# Supplementary material for: Perceived barriers and facilitators of initiation of behavioral weight loss interventions among adults with obesity: a qualitative study
Source: BMC Public Health. 2018 Jul 11;18:854. doi: 10.1186/s12889-018-5795-9 (PMC6042474; doi:10.1186/s12889-018-5795-9)
Supplement: Supplementary file 1 — Qualitative focus group/interview moderator script. This is the script used to moderate the focus groups and interviews conducted for this study. (PDF 348 kb) [file 12889_2018_5795_MOESM1_ESM.pdf]

Focus group interview script

I'd like to thank you for taking time to participate in today's focus group. The purpose of our meeting is to talk about the decision to use or not use a program to help with weight loss. One way to best help individuals make a decision about use of a program is to learn directly from people who have used or not used treatment.

Before we get started, I want to assure you that we will keep your individual responses confidential, and we ask that you do the same. Now I'll go over a few ground rules (these go on a chalkboard or flip pad).

1. Please say **exactly** what **you** think. Don't worry about what I think or what your neighbor thinks.
2. Talk about **YOUR** experience and feelings, and **not** about what you have heard **others** say about these issues.
3. Express **your** opinions, but do not argue with other participants.
4. This discussion is being taped, so please speak up and speak clearly.
5. Let's try to have only 1 person talking at one time. I will try to assure that everyone gets a turn to share their opinions.
6. Finally, we're here to have fun. I hope everyone will leave this group having had a worthwhile experience.

Treatment non-initiator

All of you have made an attempt to lose weight in the past year, and you have done so without enrolling in a structured program to help. We want to find out what your thoughts are about programs for helping people with weight loss, and what led to your decision to work on losing weight on your own, without a program.

To get started, I'd like to go around the table and have each person tell us your name and a little bit about why you decided to participate in this discussion.

When you speak, please say your first name only so that we'll know who is talking when we transcribe the audiotape.

- What led you to decide to try to lose weight?

I'm going to ask your thoughts specifically about what we call a behavioral weight loss treatment. When I talk about a behavioral weight loss treatment, I'm talking about a treatment that includes three components:

1) Education and recommendations for **nutrition**.
2) Education and recommendations for **PA.**
3) "Skills training." (Teaching of **strategies** to help you make changes). For example, tracking your food or physical activity with a diary or mobile phone app, or practicing mindfulness.

- Behavioral weight loss treatment programs are often delivered in one of three ways: in-person groups, individual by phone, or individual by internet. Now, I'm going to ask about your thoughts on using a behavioral weight loss treatment, and I will ask you about those 3 different ways it could be delivered.
- First, I want to get your thoughts on a group format.

Group: This would be a program that meets regularly in person in a group with other individuals working on losing weight. Someone with training in weight loss would lead the group. For example, this could be a program offered through duke that is a research program, or it could be a commercial program, such as weight watchers.

- To what extent have you considered joining a group-based program?
- What, if anything, do you think it would take for you to decide to use a group-based program?

(probes to be used if not brought up by group)

Tell me about any concerns related to attending this group that you might have about:

- …. being embarrassed to discuss weight?
    - What would it take to overcome that?
- … having to interact with others.
    - What would it take to overcome that?
- …being able to find the time to participate or fitting it into your schedule?
    - What would it take to overcome that?
- … the cost.
    - What would it take to overcome that?
- … those around you, such as family and friends, not being supportive.
    - What would it take to overcome that?

- … a program might ask you to do things you didn't want to do, such as eat foods you didn't want to eat or exercise more than you wanted to.
    - What would it take to overcome that?

- … who would be leading the program.
    - What would it take to overcome that?

Telephone-based: Now I want to hear about what things might make you use or not use a telephone-based weight loss program. When I refer to a telephone-based weight loss program, I am still referring to a program that includes nutrition, PA, and skills training, but one that is delivered over the phone, 1-on-1 with a trained professional.

Internet-based: Lastly, I now I want to hear about what things might make you use or not use an internet-based weight loss program. When I refer to an internet-based weight loss program, I am still referring to a program that includes nutrition, PA, and skills training, but one that is delivered over the internet. This program would involve interacting with a trained professional via the internet, and include personalized feedback.

Treatment initiator

All of you have enrolled in some sort of program to help with weight loss. We want to find out what led you to enroll in a program. When we talk about a weight loss program here, I would like you to specifically focus on the program that you joined in the past 1-2 years. For some of you this is Weight Watchers, for others it is the VA's MOVE program, and for some it may be a program that you do with your doctor or a nutritionist.

Although you might have had many reasons for wanting to lose weight, we are really going to focus in not on why you wanted to lose weight, but more on why you decided to use a program to help you lose weight, rather than try to lose weight on your own.

To get started, I'd like to go around the table and have each person tell us your name, what program you enrolled in. When you speak, please say your first name only so that we'll know who is talking when we transcribe the audiotape.

- First, what made you decide to join the weight loss program that you joined?
    - (*probe if not brought up*: Were there any particular triggers that led you to use that program. For example, you saw a commercial, your doctor recommended it, or a friend talked about it).

- The benefits of starting a program can vary from person to person. What are some things you thought might be helpful or beneficial for you about starting a program?
  (probes to be used if not brought up by group)
    - Tell me about your thoughts on learning more about nutrition. In what way was learning more about nutrition a factor in deciding to attend?
    - Tell me your thoughts about learning more about physical activity as part of a program. In what way was that a factor in deciding to attend?
    - Tell me your thoughts about the social support available in a program. In what way was having support from people a factor in deciding to attend?
    - Some people say that being involved in a program can provide motivation and inspiration to keep working on weight. In what way was that a factor for you?

    - To what extent did you consider using any other weight loss programs? What made you decide to join the one you did?
    - To what extent did you consider doing an internet-based program?

- Different things can get in the way of starting a weight loss program. What things, if any, did you have to overcome before joining a program?
  (probes to be used if not brought up by group)
    - Tell me about any concerns you had about being embarrassed to discuss weight.
    - Tell me about any concerns you had about having to interact with others as part of a program.
    - Tell me about any concerns you had about being able to find the time to participate in a program?
    - Tell me about any concerns you had about those around you, such as family and friends, not being supportive of you going to a program.

- Tell me about any concerns you had that a program might ask you to do things you didn't want to do, such as eat foods you didn't want to eat or exercise more than you wanted to.
  WHAT MADE YOU FINALLY OVERCOME THAT?
